# Pure Chitosan Biomedical Textile Fibers from Mixtures of Low- and High-Molecular Weight Bidisperse Polymer Solutions: Processing and Understanding of Microstructure–Mechanical Properties’ Relationship

**DOI:** 10.3390/ijms23094767

**Published:** 2022-04-26

**Authors:** Flor Estefany Bentley, Renaud Passieux, Laurent David, Anayancy Osorio-Madrazo

**Affiliations:** 1 Laboratory for Bioinspired Materials BMBT, Institute of Microsystems Engineering IMTEK, University of Freiburg, 79110 Freiburg, Germany; estefany.bentley@imtek.uni-freiburg.de; 2 Freiburg Materials Research Center FMF, University of Freiburg, 79104 Freiburg, Germany; 3 Freiburg Center for Interactive Materials and Bioinspired Technologies FIT, University of Freiburg, 79110 Freiburg, Germany; 4 Laboratoire Ingénierie des Matériaux Polymères IMP, CNRS, Université Claude Bernard Lyon 1, INSA de Lyon, Université Jean Monnet, UMR 5223, Univ Lyon, F-69622 Villeurbanne, France; renaud.passieux@univ-lyon1.fr

**Keywords:** chitosan, fiber spinning, crystallinity, short polymer chains, polysaccharide X-ray scattering, textile applications

## Abstract

Natural polymers, as extracted from biomass, may exhibit large macromolecular polydispersity. We investigated the impact of low molar mass chitosan (LMW, DP_w_~115) on the properties of chitosan fibers obtained by wet spinning of chitosan solutions with bimodal distributions of molar masses. The fiber crystallinity index (*CrI*) was assessed by synchrotron X-ray diffraction and the mechanical properties were obtained by uniaxial tensile tests. The LMW chitosan showed to slightly increase the crystallinity index in films which were initially processed from the bimodal molar mass chitosan solutions, as a result of increased molecular mobility and possible crystal nucleating effects. Nevertheless, the *CrI* remained almost constant or slightly decreased in stretched fibers at increasing content of LMW chitosan in the bidisperse chitosan collodion. The ultimate mechanical properties of fibers were altered by the addition of LMW chitosan as a result of a decrease of entanglement density and chain orientation in the solid state. An increase of crystallinity might not be expected from LMW chitosan with a still relatively high degree of polymerization (DPw ≥ 115). Instead, different nucleation agents—either smaller molecules or nanoparticles—should be used to improve the mechanical properties of chitosan fibers for textile applications.

## 1. Introduction

While chitosan is known for numerous advantageous biological properties such as antibacterial activity [1,2], antioxidant properties [3], biocompatibility [4,5], bioresorption [6,7], and can constitute many physical forms including hydrogel and fiber structures [8,9,10], its use as a textile product is still limited by the poor mechanical properties of chitosan fibers. In fact, textile fibers are commonly subjected to high stresses and strains during industrial knitting or weaving processes [11,12]. In addition, pure natural materials can meet biodegradability criteria, which motivates new discoveries and developments of bio-based textile fibers [13]. In this view, increasing the mechanical resistance of chitosan fibers could be an outbreak, opening the road to the production of knitted medical devices such as bioresorbable hernia meshes, antibacterial and antifungal wound dressings, sutures treads, vascular scaffolds, healthcare/hygiene products, among other fiber biomedical applications [14].

Such an objective of producing chitosan monofilaments for knitted fabrics has led to many studies attempting to increase the mechanical properties of wet spun chitosan fibers [15,16]. First studies were performed to optimize the process parameters such as the repartition of drawing ratio [17], the drying conditions [18], or the coagulation step [17,19]. Other studies were performed to optimize the composition of the collodion (solution to be extruded for further spinning) in terms of chitosan concentration [20], solvent composition, and nature, such as the use of ionic liquids [21] and incorporation of covalent crosslinking agents e.g., glyoxal [22], epichlorohydrin [23] or glutaraldehyde [24]. Recently, knittable fibers were obtained by adding copper (Cu^2+^) cations in the collodion composition in order to complex and bridge chitosan chains after coagulation and neutralization [15]. Such a strategy was shown to improve the mechanical and antibacterial properties, while preserving biocompatibility for the optimal compositions.

Another classical route to obtain improved chitosan fibers is to mix chitosan with other polymers [16,25,26]. Fibers of chitosan mixed with synthetic polymers such as PVA allowed researchers to produce fibers with good mechanical properties [25], but these may not be fully bioresorbable, biocompatible, or bioactive. Other studies focused on the production of fully bio-based and resorbable fibers. Chitosan was mixed with cellulose in a common solvent, LiOH/KOH/urea, and coagulated in phytic acid to generate H-bond interactions between both polysaccharides [16]. Chitosan was also used as a coating on preformed alginate fibers to associate the bacteriostatic behavior of chitosan and the mechanical performance of alginates [26]. It has been also explored the incorporation of polysaccharide nanofillers to improve the mechanical properties of chitosan materials [5,8,10,27,28,29,30]. For instance, the addition of chitin nanofibrils [31] or cellulose nanofibers [8] led to fibers with improved properties, with the optimum of the filler concentration found to be close to 0.3 and 0.4 wt%, respectively.

Polymer crystallites could also be considered as reinforcing nanofillers or physical crosslinkers [32,33,34,35,36,37,38]. A prominent example is the silk. Many efforts have aimed to develop useful wet spinning processes for silk fiber formation, including approaches where sericin plays an important role in the molecular orientation and thereby the re-crystallization process of fibroin [39,40]. It has been demonstrated that a high content of β-sheet crystals in silk plays a major role in the outstanding fiber mechanical performance and also leads to low degradation rates. Indeed, findings have demonstrated that it is possible to enhance the mechanical properties and control the degradation of silk fibers by tuning the content of β-sheet structure for specific applications [41]. In the case of chitosan, some attempts have been made to understand and use its crystallization to improve chitosan biomaterials’ mechanical performance [42,43,44]. Desorme et al. [45] induced crystallization into an anhydrous allomorph by adding 1,2 propanediol in the collodion [46,47]. Many other works aimed at increasing the crystalline chitosan ratio [43,44]. Since the drawing ratio is generally known to increase the crystallinity in melt spinning processes [48], its impact on crystallinity during the wet spinning of chitosan was also explored. The influence of the drawing ratio on mechanical properties seems to be essentially due to the crystallites’ orientation induced by drawing [45]. Then, the crystallinity index mainly depended on the hydration conditions during neutralization of the chitosan hydro-alcoholic collodion [47]. Crystallization can theoretically be promoted by increasing the mobility of the chitosan chains. This can be achieved by decreasing the concentration of the collodion [45]. Nevertheless, the pre-fiber (extruded hydrogel macrofilament after neutralization) might be too fragile to be efficiently stretched and thereby induce a high crystalline orientation. Another way to increase chains’ mobility is by decreasing the molar mass of chitosan [49,50], but only a few experimental studies have been performed in this context [45]. Due to the molar mass decrease, an increase of crystallinity was observed, but at the cost of a worsening of the mechanical properties, which was related to the decrease of polymer entanglement density. According to simulations by Triandafilidi et al. [51], the coexistence of chitosans with small and large molar masses should have a synergistic effect on crystallization. The large chains should help to orient the molecular network during extrusion and serve as a nucleation point for the small chains. In addition, large chains may play the role of macromolecular inter-crystalline bridges or tie together molecules between nanofibrils of the fiber microstructure. Thus, it is expected that this strategy could improve the mechanical properties of the yarns: low molar mass chains help in the formation of crystals while the high-molar masses could ensure high mechanical properties by participating in several crystallites (physical cross-linking).

The objective of this work was to study the impact of the addition of short chitosan chains in the fiber spinning collodion on the structural and mechanical properties of chitosan monofilament fibers for textile and biomedical applications.

## 2. Results

### 2.1. Chitosan Molecular Weight Evolution after Depolymerization

The preparation of low-molecular weight chitosan (LMW) was performed by depolymerization of a high-molecular weight chitosan (HMW) with a low degree of acetylation (DA = 2.5%). A yield of 85% was obtained in the depolymerization by nitrous deamination reaction in mild conditions. The characterized number-average ($M_{n}$) and weight-average ($M_{W}$) molar masses and dispersity *Đ* of the starting HMW and LMW chitosan obtained after depolymerization, are displayed in Table 1 (see also Section 4. Materials and methods). The achieved molar masses agree with those reported in the literature [52], when using molar ratios of glucosamine to sodium nitride (*r* = *n*(GlcN)/*n*(NaNO_2_)) close to 20 in the depolymerization of chitosan by nitrous deamination at room temperature.

### 2.2. Rheological Behavior of Mixture Solutions of Low- (LMW) and High-Molecular Weight (HMW) Chitosans

**Figure 1:**
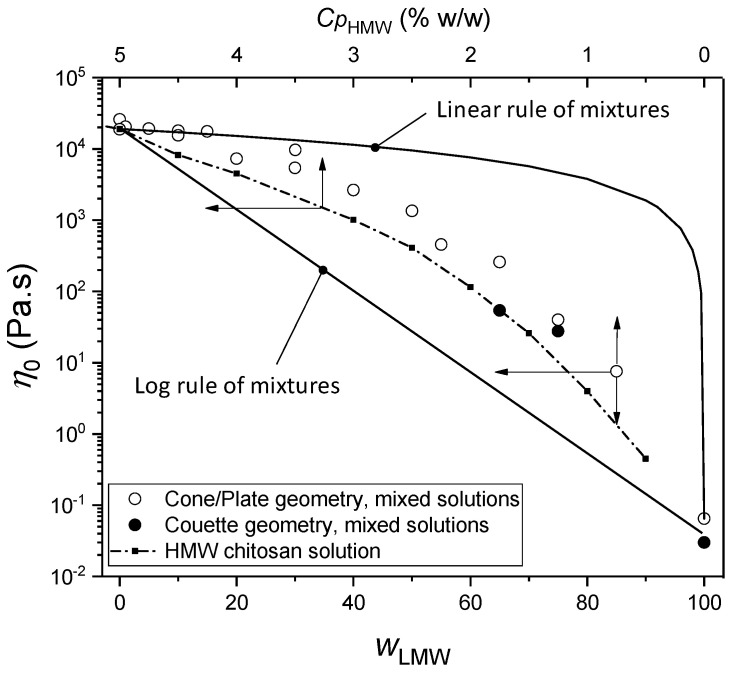
Newtonian viscosity $\eta_{0}$ of collodions consisting of a mixture of LMW and HMW chitosans at a constant total concentration *C*_p_ of 5% *w*/*w*, with varied LMW weight fraction $w_{LMW}$ (◯): Cone/Plate viscosity measurements for mixed collodions. (⬤): Viscosity obtained after rheological measurements in Couette geometry. The dashed line displays the evolution of Newtonian viscosity measured for the mono-modal HMW chitosan alone at different concentrations. The similarity in the evolution of the viscosity for mixes and for the HMW chitosan solutions evidence that the viscosity is mainly governed by the entanglement density imposed by the HMW chitosan. Thus, the evolution of viscosity in the mixtures mainly results from a dilution effect of the HMW and residual entanglements of the LMW with HMW. Solid lines display the predictions of the two mixing rules given in Equations (1) and (2).

The viscosity (η) vs. shear rate ($\dot{\gamma }$) i.e., flow diagrams of viscous chitosan solutions containing low and high-molecular weight chitosans display a plateau value at low shear rates corresponding to the Newtonian viscosity, as well as the shear-thinning behavior, typical of chitosan solutions when the shear rate increases [5,8,10,53]. The latter behavior relates to the disentanglement of the polymer chains and the evolution of the initial isotropic network towards an oriented macromolecular structure [31,54]. As a general trend, the addition of low-molecular weight (LMW) chitosan to high-molecular weight (HMW) chitosan, maintaining a constant polymer concentration, significantly decreases the viscosity. Figure 1 shows the evolution of the zero-shear viscosity with the increase of the LMW chitosan content, at a constant chitosan concentration *C*_p_ = 5%. In the LMW weight fraction range from 1 to 30%, the collodions remained highly viscous and spinnable, exhibiting viscosities above 10^4^ Pa.s. In our conditions, at pH = 4.5, acid hydrolysis of chitosan chains was shown to have negligible effects by measuring the evolution of viscosity over 2 days [55]. In the whole composition range however, the substitution of HMW by LMW chitosan induces a significant decrease in the viscosity. This evolution can be framed by the result of two phenomenological mixing rules [56], namely the linear and logarithmic mixing rules (Equations (1) and (2)):

*η*_0_ = *w_LMW_ + w _HMW_* ∗ *η_HMw_* (1)

*Ln(η*_0_) = *w_LMW_* ∗ *Ln(η_LMW_) + w_HMW_* ∗ *Ln(η_HMW)_* (2)

where *w_LMW_* and *w_HMW_* are the weight fractions, and *η_LMW_* and *η_HMW_* are the viscosities of LMW and HMW chitosan solutions (at a concentration of 5% *w*/*w*) respectively. Specific molecular interactions between the mixed components may be evidenced when the experimental viscosity of the mixture exceeds the prediction of Relation (1) [57]. Such behavior is not expected here in the mixture of two polyelectrolytes with same chemical structure. Equation (2) and its derivations can also be used as an upper bound value to evidence non-ideal mixing effects in polymer solutions, for the prediction of the evolution of solution viscosities with polymer concentration [56]. In this work, we address the case of chitosan chains that are highly extended as polycationic polyelectrolytes. The resulting viscosities, exceeding the ideal prediction of Equation (2), are related to the high entanglement density. The evolution of the Newtonian viscosity of the mixtures is in fact quite similar to that of the solutions containing the HMW component only, accounting for its dilution effect (see dashed line in Figure 1), but the viscosities of the mixtures stay significantly higher than the HMW reference. Thus, the LMW component in the mixture is thus contributing to the entanglement network, although in the pure LMW system, auto-entanglement between short chains is rare and yield a viscosity at *C*_p_ = 5%, which is almost six orders of magnitude lower than the high molar mass component.

Concerning now the ‘spinnability’ of chitosan solutions, a viscosity of ~850 Pa.s was previously reported to be the minimum value for fiber formation from HMW chitosan dopes [45]. Applying the same criterion to the mixtures of this, the composition for spinnability is thus limited to the range from *w_LMW_* = 0 up to 60%. In the following, we will focus on the composition range from *w_LMW_* = 0 to 30% for easy processing. As a general trend, the incorporation of low molar masses induces a decrease in viscosity of the HMW/LMW mixture in comparison with the 100% HMW collodion at 5%. Low molar mass chitosan could be used as a processing aid to maintain increase the overall chitosan concentration reducing viscosity, especially if the LMW addition does not induce a significant worsening of the mechanical properties of the yarns.

### 2.3. Effect of Low-Molecular Weight (LMW) Chitosan Content on the Crystallinity of Chitosan Films

**Figure 2:**
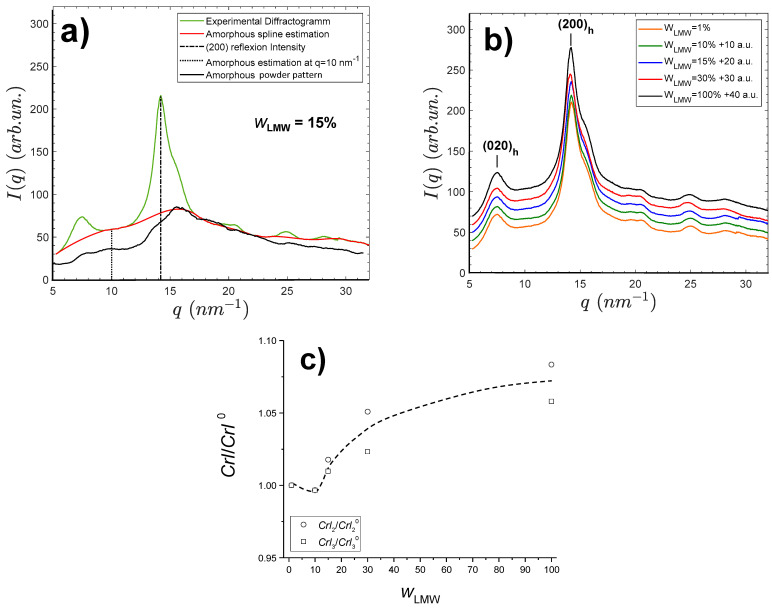
Wide angle synchrotron X-ray scattering patterns of films processed from mixed LMW and HMW chitosan aqueous solutions. (**a**) Procedures for the calculation of crystallinity index (CrI) applied to the 10% LMW chitosan-containing film diffractogram (green), by using the WAXS pattern of an experimentally obtained chitosan amorphous sample (black, $CrI_{3}$), by estimating the amorphous contribution by a cubic spline calculated from amorphous regions of the diffractogram (red, $CrI_{2}$), and by simply using the method by Focher et al. [58] relation with the intensity of the (200)_h_ reflection and that at *q*~10 nm^−1^ (dot line, $CrI_{1}$). (**b**) WAXS patterns of films containing different LMW chitosan contents $w_{LMW}$. Curves are shifted for clarity as indicated. (**c**) Normalized plots for the evolution of crystallinity indexes $CrI_{2}\;$ and $CrI_{3}\;$ with weight fraction of LMW chitosan. The normalization is performed with crystallinity indexes at $w_{LMW}$ = 1%.

Films made by water evaporation of the aqueous mixtures of the LMW and HMW chitosans and further neutralization with NaOH were analyzed by synchrotron wide angle X-ray scattering (s-WAXS). The results are shown in Figure 2. All film samples exhibit a semicrystalline morphology involving the hydrated allomorph, displaying the (200)_h_ family of planes at $14.2{\;\mathrm{nm}}^{-1}$ and the (020)_h_ planes at about $7.156{\;\mathrm{nm}}^{-1}$ [42,43]. From these X-ray scattering patterns, crystallinity indexes of the films were calculated according to three different procedures, as shown in Figure 2a. A first crystallinity index $CrI_{1}$ was obtained from the Focher et al. [58] relation: $CrI_{1}=\frac{I\left(200\right)-I\left(q=10nm^{-1}\right)}{I\left(200\right)}$. A second crystallinity index $CrI_{2}$ was deduced from the estimation of the area ratio of the crystalline peaks over the total area of diffractogram: $CrI_{2}=\frac{A_{total}-A_{am}}{A_{total}}$, with the amorphous scattering pattern estimated as a cubic spline using different scattering domains, hypothesized to be mainly due to the contribution of the amorphous phase, i.e., *q* = 5–5.3 nm^−1^; *q* = 9.3–11.2 nm^−1^; *q* = 17.3–19.1 nm^−1^; *q* = 21.95–23.7 nm^−1^; *q* = 26.3–26.85 nm^−1^; *q* = 30.2–32 nm^−1^. At last, $CrI_{3}$ estimation was based on the use of the experimental WAXS pattern of an amorphous chitosan sample as reported by Osorio-Madrazo et al. [43] (Figure 2a); hypothesizing this amorphous structure of chitosan will be representative of the amorphous phase within semicrystalline chitosan material. Briefly, the diffractogram of the amorphous sample was adjusted by a multiplicative coefficient to the diffractogram obtained for the semicrystalline film sample, in the scattering vector q range from 17 to $18{\;\mathrm{nm}}^{-1}$. The $CrI_{2}$ calculation was then performed from the area ratio, using the trapeze integration method in the scattering vector q range from 5 to $32{\;\mathrm{nm}}^{-1}$. The actual amorphous contribution might result as an intermediate value between those obtained by the cubic spline $(CrI_{2}$) and the experimental amorphous diffractogram $(CrI_{3}$) methodologies.

In Figure 2b, the films obtained with different LMW contents display similar diffractograms. The sharpest (200)_h_ peak is observed for the film prepared from pure low-molecular weight (LMW) chitosan ($w_{LMW}$ = 100%). Table 2 shows the obtained *CrI* values for films of varied LMW chitosan weight fractions $w_{LMW}$.

The values of $CrI_{1}$ did not show a clear trend, but analyzing further the finer $CrI_{2}$ and $CrI_{3}\;$ crystallinity indexes revealed an increase of crystallinity with $w_{LMW}$. The CrI values obtained at $w_{LMW}=$1% were used for normalization, as shown in Figure 2c. Such a crystallization increase can be related to a higher mobility of the short chitosan chains. In addition, using an approximation of the Scherrer equation $L_{hkl}=\frac{2\pi }{\Delta q_{hkl}}$, an apparent crystal width $L_{hkl}$ can be determined using an estimation of the full width at half maximum (FWHM) $\Delta q_{hkl}$ of the (200)_h_ and (020)_h_ reflections. For the film consisting of pure LMW chitosan $\left(M_{W\;}=19\;\mathrm{kg}\cdot {\mathrm{mol}}^{-1};\;w_{LMW}=100\%\right)$, we found a value of $L_{200}$ close to 5 nm and $L_{020}$ close to 4.5 nm. Similar values were observed for a depolymerized chitosan sample with a higher *M*_W_ = $61\;\mathrm{kg}\cdot {\mathrm{mol}}^{-1}$, showing that the size of chitosan crystallites is not significantly impacted by the chitosan molecular weight in that polymerization degree range.

The increase of the fraction of low-molecular weight chitosan LMW induces an increase in the polymer crystallinity indexes in the films, especially for LMW percentages above 15%. This increase should be due to a higher mobility of the short chitosan chains when gelation occurs, since crystallization is known to be triggered by neutralization of the solutions, leading to chitosan hydrogels with ordered chitosan chains, which can yield semicrystalline films after drying [59]. The crystallization conditions were chosen to be quite similar in the spinning process, where neutralization is performed first for the production of a semicrystalline hydrogel macrofilament that can be further stretched and finally dried. Thus, it is interesting to investigate whether an increase in LMW concentration also promotes the polymer crystallization in LMW/HMW spun fibers and whether the presence of the LMW component has a positive influence on the mechanical performance of chitosan fibers.

### 2.4. Morphology and Structure of Spun Fibers Containing Low- (LMW) and High-Molecular (HMW) Weight Chitosans

**Figure 3:**
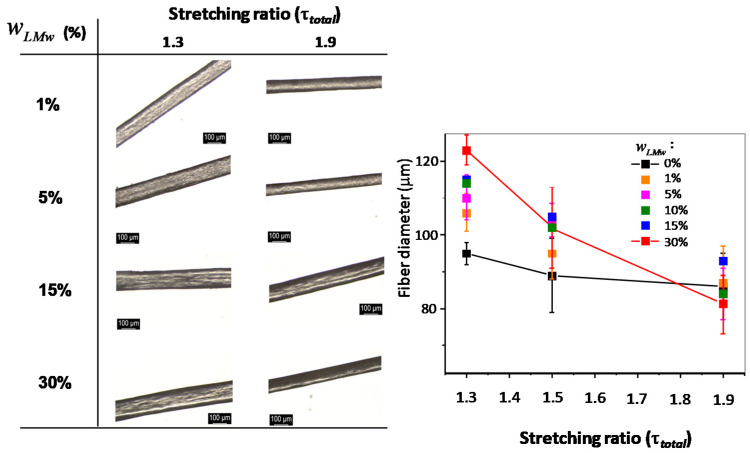
Optical microscopy images (left) and main diameter (right) of spun fibers processed with collodions containing different mass fractions of LMW chitosan $w_{LMW}$, by using different total stretching ratios $\left(\tau_{total}\right)$ of 1.3 and 1.9. Scale bar: 100 μm. The photo on the top-right shows an example of fibers of $w_{LMW}=5\%$ collected on a take-up spool.

**Figure 4:**
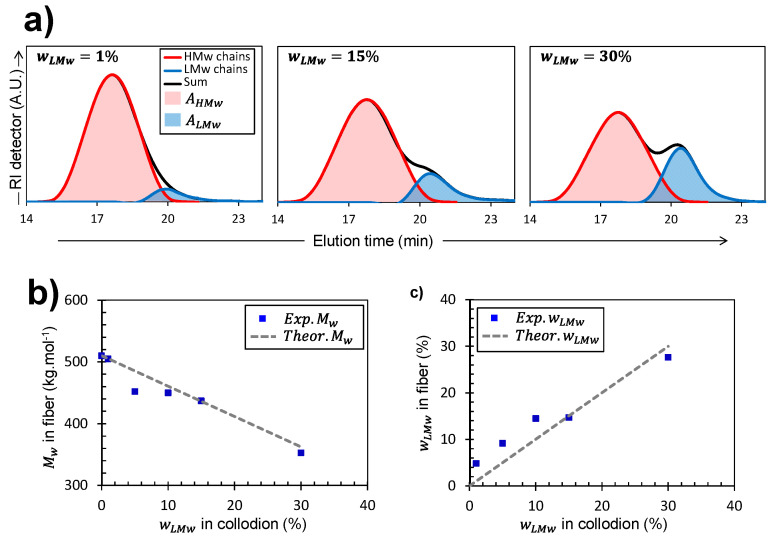
(**a**) SEC/MALLS analyses showing the high- (HMW) and low-molecular weight (LMW) chitosan chains’ distributions obtained in fibers produced from collodions containing different LMW fractions $w_{MLW}$, namely 1, 15, and 30%. (**b**) Experimental weight-averaged molar masses within the processed fibers $Exp.M_{W}$ (solid squares) produced from collodions with different LMW fractions $w_{MLW}$. The experimental values are compared with the $Theor.M_{W}$ represented by the dashed line (**c**) Fractions of LMW deduced from the SEC/MALLS measurements after dissolution of fiber samples ($Exp.w_{LMW}$) vs. LMW fraction initially incorporated in the collodion $w_{LMW}$. The fraction of LMW chitosan evaluated in the fiber from the elution diagrams is close to that of the initial collodion precursor $w_{MLW}$, evidencing the absence of leakage of LMW chains in the spinning process (dashed line: first bisector).

Optical microscopy observations of the fiber surfaces with varied LMW chitosan content and processed under different spinning stretching ratios are shown in Figure 3. There is no apparent effect on the surface or physical appearance in the presence of different proportions of LMW and HMW chitosan. The diameter of the fibers decreases as the stretching ratio is increased, for all LMW content. This result is in agreement with expectations and previous experiments performed on fibers [45,60]. Nevertheless, as the LMW chitosan content increases, the diameter differences between low and high stretching ratio increases, as seen in Figure 4, whereas it is practically negligible when using a collodion of only high-molecular weight chitosan ($w_{LMW}=0$). The high-molecular weight chitosan may bring to the extruded solution a more oriented structure that is kept in the neutralized hydrogels. On the contrary, the presence of LMW chitosan may induce a faster relaxation of the orientation at the exit of the spinneret, yielding larger fiber diameters at low stretching ratios. At higher drawing ratios, all fiber diameters are close, as a result of chain orientation. Another possibility could be the leakage of short chains in the coagulation and washing baths, which could be verified by determining the chitosan molar mass distribution in the fibers after the spinning process: short chain leakage would lead to a material loss and consequently thinner fibers. To this end, the molar mass distribution of chitosan in the processed fibers was determined by size exclusion chromatography (SEC).

#### 2.4.1. Distribution of Molar Mass in Fibers Processed from Mixtures of Chitosan Collodions

The theoretical weight-average molar mass ($Theor.M_{w}$) expected in each collodion of LMW/HMW chitosan mixture can be calculated using Equation (3):

(3) $$Theor.M_{w}=\left(w_{LMW}\times M_{w}\left(LMW\right)\right)+\left(100-w_{LMw}\right)\times M_{w}\left(HMW\right)$$

where $M_{W}\left(LMW\right)$ and $M_{W}\left(HMW\right)$ are the molar masses of LMW and HMW chitosans, respectively.

SEC/MALLS analysis of the fibers (Figure 4) allowed verification of the bi- or mono-modal nature of molar mass distributions of chitosan chains present in the fibers. It also provided their experimental weight-average molecular weights $Exp.M_{W}$, which could be compared to the theoretical $Theor.M_{W}$ as shown in Figure 4b.

Alternatively, in the case of bi-disperse collodions, it was possible to reconstruct the individual molar mass distribution curves of the two chain populations when the difference in average molar masses was distinctly observed (Figure 4a). Indeed, the elution diagram can be analyzed by considering two Gaussian curves, respectively corresponding to the population distribution of HMW and LMW, as shown in Figure 4a. The areas under the two Gaussian curves can then be used to calculate the experimental weight fraction of LMW chains ($Exp.w_{LMW}$) present in the spun fibers as follows (Equation (4)):

(4) $$Exp.w_{LMW}=100\times A_{LMW}/\left(A_{HMW}+A_{LMW}\right)$$

where $A_{HMW}$ and $A_{LMW}$ are the integrated areas of the Gaussian decompositions of the elution diagram for high- and low-molecular weight contributions, respectively. Finally, the obtained experimental fraction of LMW chitosan ($Exp.w_{LMW}$) can be compared to the weight fraction initially added in the collodion ($w_{LMW}$) as shown in Figure 4c.

Measurements of $Exp.M_{w}$ in the fibers obtained from bi-disperse collodions agree well with the expected values ($Theor.M_{w}$) (Figure 4b). This shows that despite their mobility, the short chitosan chains are not released, neither in coagulation nor in the washing bath, and were mostly fully present in the processed fibers. The same conclusion can be reached studying the fraction of LMW chitosan in the fiber, evaluated from the SEC elution diagrams, which is close to that of the initial collodion precursor $w_{MLW}$ (Figure 4c) The absence of leakage of LMW chains in the spinning process may be related to their partial entanglement to the HMW chains, as deduced from the rheological analysis.

#### 2.4.2. Crystalline Microstructure and Preferential Orientation in Chitosan Fibers: Effect of Stretching Ratio

**Figure 5:**
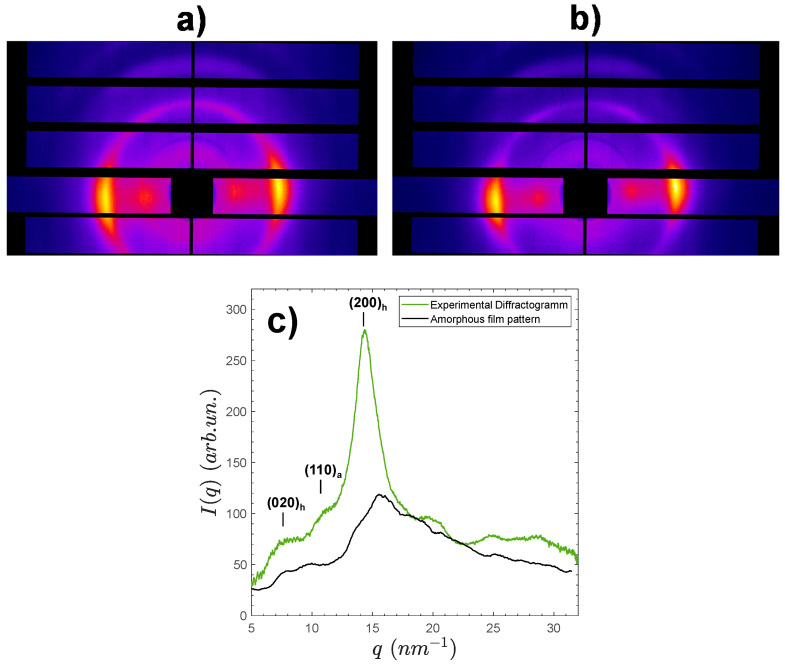
Synchrotron wide angle X-ray scattering (s-WAXS) 2D-patterns of fibers containing 1% of LMW chitosan, processed with a stretching ratio $\tau_{total}$ of (**a**) 1.3 or (**b**) 1.9. The fiber axis was almost placed vertically. Apparent Herman’s orientation factors of (**a**) −0.205 (less oriented) and (**b**) −0.238 (more oriented) were estimated for the most intense crystalline reflection (200)_h_. (**c**) radial average 1D scattered pattern ($w_{LMW}$ = 30%).

Figure 5 shows examples of two-dimensional synchrotron wide angle X-ray scattering patterns of processed fibers containing 1% of LMW, spun either with low (Figure 5a) or high (Figure 5b) total stretching ratio $\tau_{total}$.

As expected from fibers (or films) coagulated from aqueous mix collodions, all spun fibers exhibited the peaks of hydrated allomorph of chitosan, displaying the highest intensity signal of the (200)_h_ family of planes at 14.2 nm^−1^, as well as the (020)_h_ planes at about $7.4{\;\mathrm{nm}}^{-1}$, but well oriented in the equatorial sector [42]. For *CrI* measurements, 1D X-ray scattered patterns were obtained from the radial average of the 2D -WAXS images over the image center (see example for fiber with $w_{LMW}=30\%$, Figure 5c). Nevertheless, the crystalline structure of chitosan within fibers appeared more complex than in the films, since a fraction of anhydrous allomorph was also present, as evidenced by the large diffraction peak of the (110)_a_ close to *q* = 10.8 nm^−1^. The presence of the anhydrous allomorph is usually due to the crystallization of chitosan in hydrophobic conditions, such as HCl 12 M solutions as previously reported by Osorio-Madrazo et al. [43], from water/alcohol collodion mixtures [47], or is also possible from the crystallization of short chitosan chains [42,43,44]. In this work, the anhydrous component was unaffected by the fraction of LMW chains, thus it should mainly result from partial crystallization, occurring during the end of the spinning process, when the fibers are dried in hot air at 120 °C. In spite of the presence of both hydrated and anhydrous allomorphs, the crystallinity index $CrI_{3}$ of the spun fibers was calculated, as above for the films, from the ratio of an amorphous sample contribution area ($A_{am}$) to the total diffractogram area ($A_{total}$) after integration from 5 to 32 nm^−1^ (see methodology in Figure 2). Table 3 shows the resulting *CrI_3_* values obtained for all spun fibers obtained with different $w_{LMW}$ and different total drawing ratios $\tau_{total}$.

For these chitosan fibers, the influence of $w_{LMw}$ on CrI was weak and slightly decreasing, contrarily to the case of films. The impact of the stretching ratio on crystallinity was also quite limited, in contrast with many spinning processes performed on synthetic polymers in the molten state [61].

**Figure 6:**
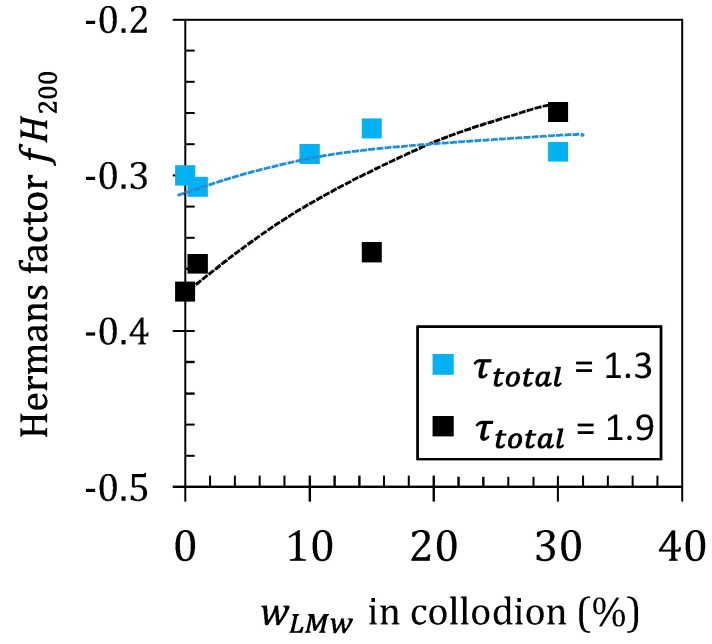
Evolution of the apparent Herman’s orientation factor ($fH_{200}$) with the LMW fraction $w_{LMw}$ in the spun fibers, estimated for processing performed at low and high total stretching ratio ($\tau_{total}$).

The influence of $w_{LMW}$ on the preferential orientation of the fibers was also evaluated (Figure 6). As shown in Figure 5, the 2D s-WAXS images of the high stretching ratio samples (e.g., Figure 5b) showed a higher intensity in the meridional spots (crystallites oriented with the normal of (200)_h_ planes perpendicular to fiber axis) in comparison with the 2D patterns of fibers processed with lower stretching ratios (e.g., Figure 5a). Thus, to quantify the anisotropy of the crystallites in the fibers, the apparent Herman’s orientation factor $fH_{200}$ was determined. To this end, using the signal of the chitosan crystallographic peak (200)_h_ at a fixed *q*_(200)_ value, the average of cos^2^(φ) is calculated, φ being the angle between the vertical fiber axis and the normal of the (*hkl*) planes [62]. Equations (5) and (6) display the classical but phenomenological relations used to determine an approximation of the Herman’s orientation factor $fH_{hkl}$ from 2D X-ray scattered intensity data:

(5) $$fH_{hkl}=\frac{3}{2}\;\langle \cos^{2}\varphi \rangle_{hkl}-\frac{1}{2}$$

(6) $$\langle \cos^{2}\varphi \rangle_{hkl}=\frac{\int_{0}^{2\pi }I_{hkl}\left(\varphi \right)\cos{\left(\varphi \right)}^{2}\sin\left(\varphi \right)d\varphi }{\int_{0}^{\pi /2}I_{hkl}\left(\varphi \right)\sin\left(\varphi \right)d\varphi }$$

where $I_{hkl}\left(\varphi \right)$ is the reflected intensity of the (*hkl*) plane family, measured at the azimuthal angle φ (φ=0 in the fiber axis) and (hkl)=(200).

Thus, a value of $fH_{hkl}=0$ corresponds to an isotropic material, $fH_{hkl}=-0.5$ corresponds to (hkl) planes with normals perfectly oriented in the equatorial axis, while $fH_{hkl}=1$ corresponds to planes that are normal and perfectly oriented in the meridional axis (i.e., fiber axis) [17]. In Figure 6, it can be observed that for the low stretching ratio ($\tau_{total}=1.3$), $fH_{200}$ remains almost constant and only slightly increases with $w_{LMW}$. When the fibers are processed at a higher stretching ratio ($\tau_{total}=1.9$), the orientation of the fibers is stronger, but the incorporation of LMW chitosan significantly decreases the orientation of the crystalline phase in particular at high $w_{LMW}$ values. Thus, shorter chains are efficient in suppressing orientation effects during the spinning process. Such chains are less entangled in the polymer network. They are also more mobile and relax faster after extrusion. Thus, increasing the short chain fraction results in a decrease of fiber orientation since a fraction of chains will not participate in crystallite alignment by inter-crystalline chain extension.

### 2.5. Mechanical Properties of Chitosan Spun Fibers for Textile Applications

**Figure 7:**
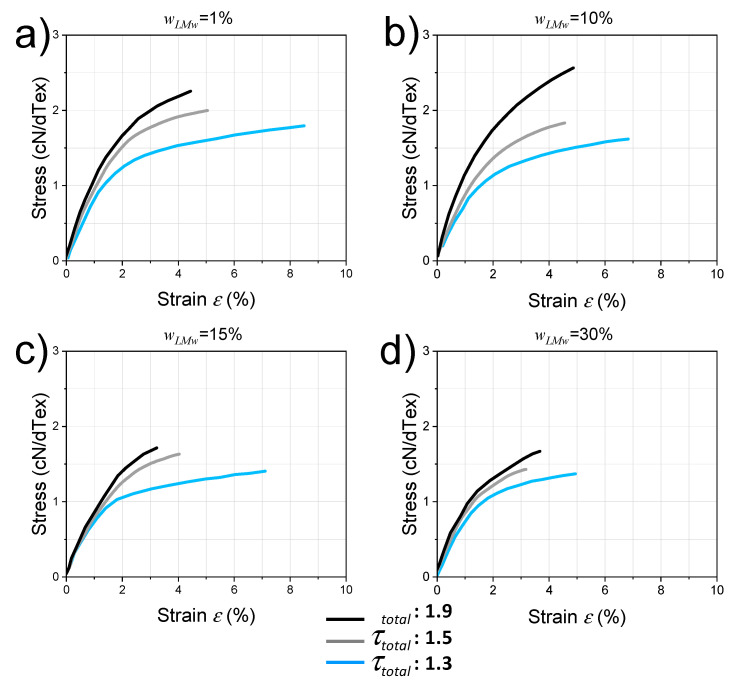
Typical nominal stress (in Textile units cN/dTex) vs. nominal strain curves for spun fibers with different LMW chitosan contents $w_{LMW}$, and for stretching ratios $\tau_{total}$ = 1.3, 1.5 and 1.9. (**a**): $w_{LMW}$ = 1%; (**b**): $w_{LMW}$ = 10%; (**c**):$\;w_{LMW}$ = 15% and (**d**):$\;w_{LMW}$ = 30%.

Table 4 displays the values of Young’s modulus E and strain-at-break $\varepsilon_{b}$, deduced from stress–strain curves of fibers in uniaxial tensile testing (Figure 7). Again, these mechanical evaluations were performed on fibers with different LMW chitosan contents and processed under stretching ratios of 1.3 and 1.9.

As a general trend, the strain-at-break decreases with stretching ratio and LMW content. The Young’s modulus increases with drawing ratio from $\tau_{total}$ = 1.3 to $\tau_{total}$ = 1.9 by an average factor of 1.3 (in textile industry units) and 1.7 (in GPa). For $w_{LMW}$ = 30%, both fibers at $\tau_{total}$ = 1.3 and 1.9 exhibit similar crystalline contents *CrI* (Table 3) and similar crystalline orientations (Figure 6). Thus, the increase of tenacity by drawing appears related to the orientation within the main amorphous phase with a possible secondary role of the hydration state and entanglement density.

In terms of fibers for textile applications, the stress-at-break of the fibers was practically calculated using textile tenacity *T*_e_ = *F_b_*/*Y*_T_, where *F_b_* is the applied uniaxial force at break (usually expressed in cN) and *Y*_T_ is the yarn titer (equivalent of the linear mass, expressed in dTex; 1 dTex = 1 g/10,000 m). The use of this textilian unit (cN/dTex) is also convenient to calculate the textilian stress and elasticity modulus since it permits us to avoid dividing by the yarn section, which can be inaccurate for some textiles such as multi-filaments. Figure 7 shows the tensile behaviors of the fibers i.e., textile stress vs. nominal strain obtained for each of the LMW chitosan contents and for different total stretching rates 1.3, 1.5, and 1.9. All systems exhibit a linear domain where the Young’s modulus is well defined and a plastic behavior before rupture. For a given composition, the tenacity (stress at rupture) of the fibers is systematically lower for a drawing ratio of 1.3 and higher for the drawing ratio of 1.9.

## 3. Discussion

### 3.1. Evolution of Crystalline Microstructure and Preferential Orientation in Spun Fibers, and Trade-off Strategies for the Choice of an Optimal Value for LMW Molar Mass

The increase of the crystallinity index ($Cr_{3}$) with the addition of the LMW chitosan observed in neutralized and dried films (Table 2) could not be translated on spun fibers where a slight decrease of crystallinity was observed. (Table 3). This discrepancy, and the specific crystalline microstructure resulting in a mixed (hydrated/anhydrous) allomorph after fiber drying, at 120 °C, reveals different crystallization processes during film forming and fiber spinning. Thus, the role of the short chains is different between film and fiber processing. The higher mobility of the short polymer chains may result in a higher nucleation and growth of crystallites in films. However, during fiber spinning, crystallization and crystalline reorientation occur by amorphous chain stretching that is limited in the case of short chains, as deduced by the increase of the orientation factor *f*_H200_ as displayed in Figure 6. The molar mass of LMW chitosan used in this work (19 kg/mol) was enough to (*i*) allow their partial entanglement with long chains, (*ii*) prevent their diffusion out of the gel in the neutralization and washing baths, and (*iii*) induce a significant decrease in the viscosity of the spinning dope (see Figure 1 for a comparison between a 5% HMW dope at $w_{LMW}$ = 0 and the viscosity of mixed dopes with $w_{LMW}$ > 10%). Meanwhile, the chains may actually be too short to participate efficiently in inter-crystalline tying of molecules that would favor crystalline orientation by hydrogel stretching. As a result, a different tradeoff value needs to be found for the optimization of the molar mass of the LMW, accounting for the processability (viscosity) of the dope, the polymer crystallization ability, and the capacity to maintain inter-crystalline junction and entanglements within the polymer network. A higher nucleation and crystallization ability might be obtained by further decreasing the length of chitosan chains i.e., by using chito-oligosaccharides (COS) with a degree of polymerization <50 (*M*_W_~8 kg/mol). Such molecules would not contribute to increasing the dope viscosity, but need to stay insoluble in the coagulation and washing baths, a property that could be met if the DP values are chosen above around DP = 35 [44,63]. Further studies are ongoing to establish the impact of COS of various degrees of acetylation (DAs) on the crystalline ratio of films and fibers. Another possibility is the use of medium chains as HMW chitosan component, if a different compromise can be found between the processability of the collodion at elevated concentrations, as well as the extensibility of the hydrogel macrofilament for chain and crystallite orientation, and the persistence of inter-crystalline tie molecules and entanglement in the solid polymer matrix. At last, the nucleation role of the short chains could be replaced or enhanced by the use of preformed polysaccharide nanocrystals (e.g., chitin or chitosan whiskers), incorporated in the collodion as a suspension [64].

Summarizing, in the chosen spinning conditions for this study, the increase in $w_{LMw}$ could not alone promote crystallization, and resulted in less orientation of the chitosan crystallites and amorphous chains (Figure 6). Shorter polymer chains contribute to a decrease in the entanglement density and thus a decrease of the molecular orientation of the chain segments between these physical crosslinks, within the amorphous phase. The presence of short chains may also have increased the molecular mobility and the relaxation phenomena occurring during the extrusion and stretching. These effects could not be balanced by the co-crystallization of the short and long chains.

### 3.2. Mechanical Properties of Mixed Chitosan Fibers for Textile Applications

**Figure 8:**
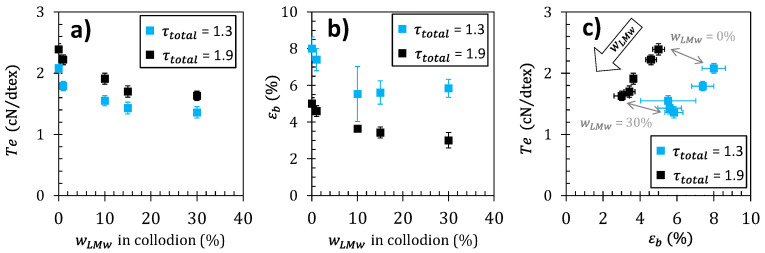
(**a**) Fiber tenacity-at-break ($T_{e}$) and (**b**) strain-at-break $\varepsilon_{b}$ vs. LMW chitosan content $w_{LMW}$, at the stretching rates ${}_{total}$ of 1.3 and 1.9. (**c**) Rupture plot for different LMW chitosan contents $w_{LMW}$ and stretching ratios. The arrow in represents the mechanical property trend associated with a $w_{LMW}$ increase.

The optimization of the mechanical properties of polymer fibers is generally a subtle task relating several processing and mechanical property parameters such as Young’s modulus, strength/tenacity, and strain-at-break. As a result, the consideration of one mechanical property vs. a unique processing parameter is generally not sufficient to establish if actual behavior achievements could be effectively obtained. As an example of multiple variable discussion, considering the stretching ratio and the composition of the chitosan dopes as processing parameters, the ultimate mechanical properties can be studied as shown in Figure 8a,b. The impact of $w_{LMW}$ is shown on the textile tenacity *T*_e_ and strain-at-break *ε_b_*. The values obtained are consistent with previously reported values of the tenacity of chitosan fibers [65]. The textile tenacity increases with stretching ratio. This is consistent with several previous literature data, reporting that improvements in the mechanical properties of the fibers are attainable by applying stretching to the fiber during the wet spinning process [17,24,66]. Tenacity decreases as the LMW chitosan content increases. Fibers with 1% LMW chitosan content have a higher tenacity (~2.2 cN/dTex), comparable to the pure high-molecular weight (HMW) chitosan fiber, while, when the LMW chitosan content further increases to 30%, the fiber tenacity decreases to ~1.4 cN/dTex. The strain-at-break also decreases with the increase of $w_{LMw}$ parameter. Thus, the introduction of LMW chitosan is detrimental to the mechanical properties, for the different stretching ratios investigated here. The rupture plot (i.e., *T*_e_ vs. *ε_b_*) is another property correlation to be analyzed as shown in Figure 8c. An improvement of ultimate property parameters would result in a shift in the upper-right quadrant of the rupture envelope draw, as grey arrows joining the rupture points obtained at different stretching ratios. Here again, the introduction of LMW chitosan induces a shift of rupture envelopes to lower *T*_e_ and *ε_b_* (Figure 8c), synonymous with a general decrease of the fiber ultimate properties. 

These trends are in agreement with the work of Triandafilidi et al. [51], modeling the crystallization of a bi-disperse polymer: short chains induce a rapid and isotropic crystallization process due to their mobility but hinder molecular orientation which compromises the increase in crystalline rate and crystalline orientation by stretching [51]. Jabbarzadeh et al. [67] also show that stretching of polymers of low-molecular weight does not induce an increase in crystallization, in contrast to the stretch applied to high-molecular weight semicrystalline polymers. This theoretically results in a decrease in tenacity and strain-at-break, and thus an overall decrease in mechanical properties upon decreasing the chain length.

#### Correlation between Mechanical Performance of Fibers and Crystalline Orientation

**Figure 9:**
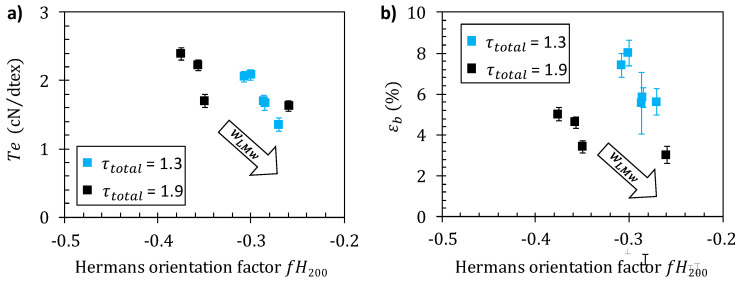
Evolution of fiber tenacity (stress-at-break) Te (**a**) and strain-at-break $\varepsilon_{b}$ (**b**) with the Herman’s orientation factor $fH_{200}$ at stretching ratios of 1.3 and 1.9, with different chitosan low-molecular weight content $w_{LMW}$.

Figure 9a confirms a close relation between fiber tenacity and crystallite orientation as revealed by the apparent Herman’s factor for the (200) planes. The values of tenacity obtained at different stretching ratios and different LMW contents fall on a common pattern. Thus, the fiber material becomes more anisotropic (*fH*_200_ decreases towards −0.5) as the stretching rate increases but an opposite trend was observed as parameter $w_{LMW}$ increases (see arrow in Figure 9a). Stretching rate increases improve tenacity for a given polymer formulation (Figure 8), but the addition of short chains decreases the mechanical strength, mainly as a result of a decrease in crystalline (and macromolecular) orientation as $w_{LMW}$ increases. Such a close relation between tenacity and orientation is in good agreement with recent results concerning the optimization of the wet spinning process of chitosan [15].

The increase in orientation also correlates with a decrease of $\varepsilon_{b}$ (Figure 9b), which is a classical result described in the literature. Nevertheless, the values of *ε_b_* obtained at different stretching ratios and different $w_{LMW}$ do not fall on a single pattern. Apparently, the strain-at-break is impacted by orientation when the chains are extended, but the presence of short chitosan chain further introduces an additional fragility mechanism particularly effective at high stretching ratios, possibly related to disentanglement of the LMW component.

## 4. Materials and Methods

### 4.1. Starting High-Molecular Weight Chitosan (HMW)

The starting high-molecular weight chitosan (HMW) was obtained from squid pen chitin and supplied by Mahtani Chitosan (CHITOSAN 114, Batch No. 20120926, Veraval, India). The HMW degree of acetylation (DA) was estimated as 2.5%, by $H^{1}$ NMR spectroscopy following the methodology of Hirai et al. [68].

### 4.2. Obtaining Low-Molecular Weight Chitosan (LMW) by Nitrous Deamination Depolymerization

Low-molecular weight chitosan (LMW) with controlled degree of polymerization (DP) was obtained by nitrous deamination. Sodium nitrite was added to a starting solution of the high-molecular weight chitosan at 4% (*w*/*v*), which was dissolved in an stoichiometric amount of acetic acid to protonate the chitosan amine groups, and considering a molar ratio r of glucosamine to NaNO_2_ of 20 (Equation (7)).

(7) $$r=\frac{n_{GlcN}}{n_{NaNO_{2}}}$$

Equation (5) defines the deamination molar ratio r where $n_{GlcN}$ and $n_{NaNO_{2}}$ are the quantities of substance (moles) of glucosamine (GlcN) residues and of sodium nitrite, respectively. The given amount of NaNO_2_ contained in a freshly prepared solution was added dropwise to the chitosan solution with magnetically stirring for 24 h at room temperature, allowing the NaNO_2_ to react with the GlcN units and to depolymerize the chitosan chains. Then, the chitosan was precipitated by adding NaOH 1 M until reaching a pH=9, then was furtherly centrifuged and washed. The centrifugation was realized at 1000 rpm for 10 min at 20 °C. While the supernatant was carefully poured into a beaker to avoid loss of coagulated chitosan, distilled water was added to the centrifugate to wash the chitosan. Washing was performed until the pH and conductivity of the supernatant was measured to be equal to that of the distilled water. Finally, the sample was dried by lyophilization, and the chitosan powder was collected.

### 4.3. Size Exclusion Chromatography/Multi-Angle Laser Light Scattering (SEC/MALLS) Characterization of Chitosan Molecular Weight 

The chitosan molecular weight was determined by size exclusion chromatography (SEC) coupled to multi-angle laser light scattering (MALLS) [43]. Chitosan solution at 0.1% (*w*/*v*) was prepared in an acetic acid/ammonium acetate buffer pH =4.5 (AcOH (0.2 M)/AcONH*4* (0.15 M)), which was used as eluent. Before SEC measurements, the solution was filtered through 0.45 μm pore size membranes (Millipore). The chromatographic equipment was composed of an IsoChrom LC pump (Spectra-Physics, Charbonnières les Bains, France) connected to a Protein Pack 200 SW column (WATERS, Saint-Quentin-en-Yvelines, France) and a TSK gel G6000 PWXL column (Merck, Saint-Quentin-Fallavier France). A Multi-Angle Laser Light Scattering (MALLS) detector DAWN DSP (Wyatt Technology, Toulouse France) operating at 632.8 nm was coupled on line to a 410 differential refractometer from WATERS (Saint-Quentin-en-Yvelines, France).

### 4.4. Preparation of Viscous Collodions Containing Low- (LMW) and High-Molecular Weight (HMW) Chitosans

Viscous solutions of low- (LMW) and high-molecular weight (HMW) were prepared for the spinning of all-chitosan yarns. High-molecular weight HMW chitosan was mixed with varied percentages of LMW chitosan, at 0, 1, 5, 10, 15, and 30 wt%, to yield mixed chitosan acetate solutions (hereafter named collodions or dopes) containing a total polymer concentration of 5% (*w*/*w*). Chitosan powder was initially swollen in distilled water for 2 h; then, acetic acid was added in a stoichiometric amount as to protonate the amine moieties of chitosan (DA=2.5%) in order to solubilize the chitosans, and the obtained mixture solution was kept under mechanical stirring overnight. Finally, viscous aqueous collodions containing LMW and HMW chitosans, and the reference chitosan solution, were obtained for further fiber spinning.

### 4.5. Shear Rheological Tests on All-Chitosan Collodions

An AR2000 rheometer (TA Instruments Ltd., New Castle, DE, USA) fitted with a cone-plate geometry (diameter: 25 mm; angle: 4°), was used to characterize the rheological behavior of the different chitosan viscous formulations at 25 °C, with a gap size of 0.116 mm and a solvent trap to prevent its drying or evaporation. The cone-plate geometry allows one to ensure a uniform shear rate throughout the sample. The analysis was performed in triplicate in continuous mode in a shear rate range from 0.005 to 1000 s^−1^. The flow diagrams, namely the plots of the steady-state shear viscosity vs. shear rate of the viscous collodions, were obtained to deduce the Newtonian viscosity in the low shear rate range plateau.

### 4.6. Films Preparation with Low- and High-Molecular Weight Chitosan Mixture Solutions

In order to investigate the influence of addition of LMW proportions to high-molecular weight chitosan on sample microstructure, films were produced under coagulation conditions similar to the spinning process as described below. The different dopes were used to create chitosan films of low- and/or high-molecular weight, with LMW chitosan fractions of 1, 10, 15, 30, and 100%. An amount of 3 g of the dope was placed in a Petri dish to obtain a solution sample of a thickness of approximately 1 mm. NaOH 1 M was added to the Petri dishes dropwise to coagulate the solutions. The Petri dish was then sealed and left for neutralization to occur for 1 h. Afterwards, they were rinsed with deionized water a minimum of 10 times, until NaOH was washed by pH measurement of the water used to rinse. Then, the solutions were left to dry in air in the Petri dishes until films were formed.

### 4.7. Wet Spinning of Chitosan Mixture Collodions

**Figure 10:**
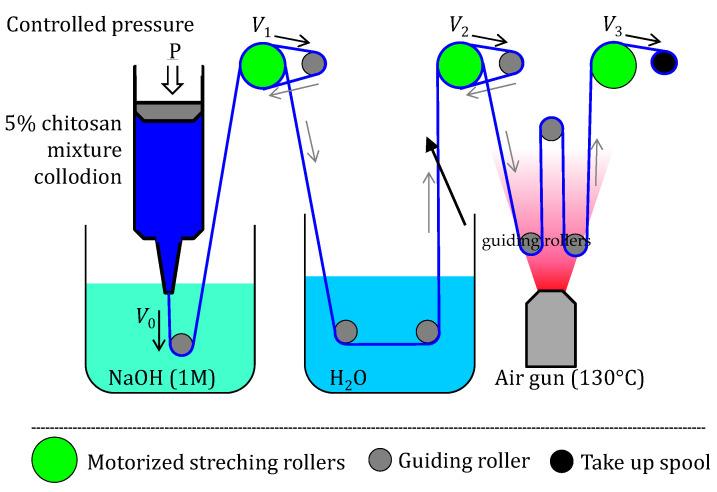
Wet gel spinning set up used to create fibers containing low and high-molecular weight chitosans, consisting of a pressurized syringe with conic needle (Nordson^®^ dispenser, Saint-Thibault-des-Vignes, France), a coagulation bath (Sodium Hydroxyde solution), 3 motorized stretching rollers (in green), which rotate at an adjustable speed to control the stretching rates, 8 guiding rollers (circles in blue/black), guiding the fiber into washing and drying zones with different stretching rates, and a final take-up spool (last black circle) where the fiber is stored once it has been stretched, washed and dried.

Viscous collodions containing mixture of LMW and HMW chitosan with compositions similar as in Table 2 were prepared. All solutions were spinnable except for the 100% LMW chitosan acetate solution—the coagulation did not produce a hydrogel strong enough as to allow gel spinning. Specifically, LMW percentages of 0, 1, 5, 10, 15, and 30 wt% were considered in the processing of fibers. The chitosan mixture was dissolved in a weak amount of acetic acid, which obtained viscous collodion that was used to create ‘pure’ chitosan fibers by wet spinning [17,69]. The experimental fiber spinning set-up is shown in Figure 10. A syringe containing the dope was attached to the air pressure clip and a spinneret cone was added at the other end. The spinneret used for all runs was a precision conic extrusion needle of inner diameter 0.41 mm (Nordson EFD, Feldkirchen, Germany), and the collodion was extruded by applying a pressure of 190 kPa. For the chitosan coagulation bath, a neutralizing solution of 1 M NaOH was used. The washing bath consisted of distilled water. The devices constituting the spinning set up are as follows [15]:

- Compressed air pumping/extrusion dispensing system (Ultimus I, Nordson EFD, Feldkirchen, Germany)
- Air heater (LHS 21S Premium, Leister, Sapelmeca Les Ulis, France))
- Power supply (ALR3003, ELC, Annecy, France)
- Rotating motors and redactors (Portescape, La Chaux-de-Fonds, Swizerland)

Overall, the set-up consisted of 8 guiding rollers, 3 stretching rollers attached to a DC motor/redactor assembly, and the final take-up spool, where the fiber is stored once it has been washed and dried (Figure 10). The drying of the fiber is a critical step and was supported by a heat gun placed in the setup before the last guiding roller, allowing heating at 130 °C (maximum temperature, measured at the closest waypoint of the fiber). The 3 stretching rollers attached to the motor have a controllable speed ($V_{1}$, $V_{2}$, and $V_{3}$) by adjusting the voltage supply to the DC motors. As a result, their speed ratios determine the stretching ratio at each of the sections of the spinning process: stretching ratio at coagulation $V_{1}/V_{0}$, stretching ratio at wash $V_{2}/V_{1},$ and stretching ratio at drying $V_{3}/V_{2}$. These 3 stretching rates were used to obtain the total stretching ratio $\tau_{total}=V_{3}/V_{0}$ as exemplarily shown in Table 5 for the fibers containing a percentage of LMW of 1%. Each of the colloidal mixtures with LMW at 0, 1, 5, 10, 15, and 30 wt% were used to create fiber samples at low (1.3), medium (1.5), and high (1.9) $\tau_{total}$ (Table 5). During the wet-spinning runs, the stretching rate at drying was the only one that was varied, to obtain the chitosan fibers, while the other two (coagulation and washing stretching rates) were kept constant (Table 5).

### 4.8. Optical Microscopy

The surface morphology and dimensions of the chitosan fibers, obtained with varied LMW chitosan concentration and under different stretching ratios, were observed under an optical microscope (LEICA M205 A, DFC450 C). The diameter was obtained by image processing.

### 4.9. X-ray Synchrotron Scattering (WAXS and SAXS)

Synchrotron X-ray scattering analyses at wide angles (WAXS) of chitosan fibers and films, obtained with different LMW weight fractions, were performed at the D2AM/BM2 beamline at the European Synchrotron Radiation Facility ESRF (Grenoble, France). The processed spun fibers were wrapped 10 times around a metal sample holder with a hollow center to allow the X-ray beam to pass through a single bundle of fibers as previously described [8,47]. The film samples, prepared as above, were placed on the hole of a sample holder allowing for X-ray scattering in transmission mode. Synchrotron wide angle X-ray scattering data were collected at a wavelength λ=0.774875 Å using a “WAXS Open for SAXS” (WOS) 2D detector from IMXPAD company (La Ciotat, France). Chromium oxide was used as standard to calibrate the scattering vector q-range, and transmission corrections and background subtraction were perfomed in the data treatment. Radial averages were finally calculated around the image center (mean center of incident beam). The empty cell signal was subtracted from the images.

### 4.10. Fiber Tensile Testing

The fiber titer was calculated from the mass of 2 m length of fiber (measured by wrapping the fiber 32 full turns around the spool of a known diameter, Ø=20 mm).

To measure the tensile properties of the chitosan fibers, a AG-X plus SHIMADZU (Noisiel, France) device was used with a 100 N load cell (SLBL-100N). A unidirectional tensile test at constant speed (50 mm/min) was set up for each sample at ambient temperature. The fiber was placed between a clamping set up 50 mm apart, which determined the effective initial length $l_{0}=50\;mm$ (then the initial deformation rate was 100%/min). The tenacity $T_{e}$ was calculated as the ratio of the applied force $F_{b}$ at rupture to the titer of the yarn *Y*t ($Te=F_{R}/Yt$), and the nominal strain ε was expressed as the ratio of the extension of the fiber respect to initial length $l_{0}$ ($\varepsilon =\Delta l/l_{0}=\left(l-l_{0}\right)/l_{0}$). Young’s modulus (E), tenacity, ($T_{e}$) and strain-at-break ($\varepsilon_{b}$) were determined from the obtained stress–strain curves, considering at least five measurement replicates (n=5) for each fiber processing condition (composition/processing), i.e., obtained under a given total stretching ratio and with a specific LMW percentage.

## 5. Conclusions

Wet spinning of pure chitosan fibers was successfully achieved by using a ‘bimodal’ dope made of a mixture of low- (*Mn* = $1.9\times 10^{4}\;g/\mathrm{mol}$, DP ~ 115) and high-molecular weight chitosan (*Mn* = $6.1\times 10^{5}\;g/\mathrm{mol}$, DP=3765) at different fractions. It was demonstrated that the mechanical and structural properties of these fibers are strongly impacted by the percentage of short chains (LMW) introduced in the dope. As the content of short chains in the mixture increases, the viscosity of the dope decreases, which might support the spinning process of collodions with higher polymer concentrations.

At preparing films, a proof of concept was established where we added shorter polymer chains (DP~115) into the chitosan solution, which enhanced chitosan crystallization.

However, in the processing of fibers, the stretching apparently limits the crystallinity in the presence of LMW chitosan. Future research could be dedicated to investigate the effect of even shorter chains, in the chito-oligomer range, and the use of oligomers with higher chitosan degree of acetylation (DA). The targeted *DP* for chitosan oligomers could be DP ~ 20−40 [70].

Further data treatment and the obtained results led us to establish the unfavorable role of the used short chains on the orientation of crystallites (and possibly the amorphous phase) in the stretching process of fibers. The addition of LMW chitosan is likely to decrease the entanglement density within the collodion but also within the hydrogel after stretching and in the final dry fiber. This decrease in entanglement density is not compensated by an increase in *CrI*, since a simultaneous decrease in tenacity and strain-at-break induced was observed with further increases of LMW content. The low molar fraction of the molar mass distribution and the polydispersity of such distribution should be carefully controlled for high demanding mechanical applications in textile industry. Further increases of the crystallinity ratio might be obtained, for example, by adding particle nucleation agents (ex: nanofibers of cellulose, chitin, or chitosan itself) or small chitosan oligomers [8,10,30].

## Figures and Tables

**Table 1:** Weight-average (*M_W_*) and number-average (*M_n_*) molar masses, polymerization degree *DP*, and dispersity *Đ* of the starting high molar mass HMW, and the low molar mass chitosan LMW obtained after depolymerization by nitrous deamination.

**Chitosans**	$M_{W}\;(g\cdot {\mathrm{mol}}^{-1})$	$M_{n}\;(g\cdot {\mathrm{mol}}^{-1})$	*DP_w_*	*Đ* $\;(M_{W}$ $/M_{n})$
HMW	$6.1\times 10^{5}\;\left(\pm 9.6\%\right)$	$4.1\times 10^{5}\;\left(\pm 6.4\%\right)$	3765	1.5 (±11.6%)
LMW	$1.9\times 10^{4}\left(\pm 1.2\%\right)$	$1.3\times 10^{4}\left(\pm 0.6\%\right)$	115	1.5 (±1.3%)

**Table 2:** Crystallinity indexes $CrI_{1}$, $CrI_{2}$, and $CrI_{3}$ obtained by different methods using X-ray diffraction patterns for chitosan films obtained with varied weight percentage of LMW chitosan $w_{LMW}$ in the precursor solutions. $CrI_{1}$ was obtained from Folcher et al. [58] $CrI_{2}$ and $CrI_{3}$ were obtained as previously reported by Osorio-Madrazo et al. [43].

$w_{LMW}\;\mathrm{in}\;\mathrm{Film}\;(\%)$	$CrI_{1}\;(\%)$	$CrI_{2}\;(\%)$	$CrI_{3}\;(\%)$
1	72.7	17.8	32.4/
10	72.5	17.4	31.4
15	72.4	17.8	31.8
30	71.8	18.4	32.6
100	73.0	19.0	33.8

**Table 3:** Crystallinity index ($CrI_{3}$) of spun fibers with increasing LMW chitosan contents, at stretching ratios $\tau_{total}$ of 1.3 and 1.9.

$w_{LMw}\;\mathrm{in}\;\mathrm{Fiber}\;(\%)$	$CrI_{3}\;(\%)$	
	$\tau_{total}\;=\;1.3$	$\tau_{total}\;=\;1.9$
0	30.7	29.7
1	30.7	29.7
10	29.3	29.2
15	29.3	29.6
30	29.4	29.1

**Table 4:** Young’s modulus E, and strain-at-break $\varepsilon_{b}$ obtained from stress–strain curves obtained by the uniaxial tensile testing of spun fibers with different contents of LMW chitosan $w_{LMw}$, using different stretching ratios $\tau_{total}$ of 1.3 and 1.9. The tensile tests were performed at 35% relative humidity.

***w*** ***_LMW_*** ** in Fiber (%)**	Young’s Modulus *E* (N/dtex; GPa)		$\mathrm{Strain}\;\mathrm{at}\;\mathrm{Break}\;\varepsilon_{b}\;(\%)$	
	$\tau_{tot}\;=\;1.3$	$\tau_{total}\;=\;1.9\;$	$\tau_{total}\;=\;1.3$	$\tau_{total}\;=\;1.9\;$
0	8.9 ± 1.1 N/dtex (11.2 ± 1.4 GPa)	11.0 ± 0.6 N/dtex (14.6 ± 0.8 GPa)	8.0 ± 0.6	5.0 ± 0.3
1	9.4 ± 0.4 N/dtex (10.9 ± 0.5 GPa)	10.6 ± 0.7 N/dtex (16.1 ± 1.0 GPa)	7.4 ± 0.6	4.6 ± 0.3
5	7.6 ± 1 N/dtex (8.5 ± 1.2 GPa)	9.9 ± 1.2 N/dtex (16.3 ± 1.9 GPa)	6.1 ± 0.6	4.0 ± 0.3
10	7.6 ± 0.9 N/dtex (7.9 ± 1.5 GPa)	11.5 ± 1.1 N/dtex (16.6 ± 0.7 GPa)	5.9 ± 1.5	3.6 ± 0.2
15	7.9 ± 0.6 N/dtex (9.7 ± 0.7 GPa)	9.7 ± 0.8 N/dtex (15.5 ± 1.3 GPa)	6.0 ± 1.1	3.4 ± 0.3
30	7.4 ± 0.8 N/dtex (8.1 ± 0.9 GPa)	9.7 ± 0.5 N/dtex (15.6 ± 0.7 GPa)	5.8 ± 0.5	3.0 ± 0.4

**Table 5:** Example of spinning stretching ratios used in experiment runs for samples containing $w_{LMW}$ = 1%. The 3 stretching rollers attached to the motor have a controllable speed ($V_{1}$, $V_{2},$ and $V_{3}$) by adjusting the voltage supply (Figure 10). Their speeds determine the stretching ratio at each of the sections of the spinning process: stretching ratio during coagulation step $V_{1}/V_{0}$, stretching ratio during the wash step $V_{2}/V_{1}$, and stretching ratio during the drying step $V_{3}/V_{2}$. These 3 stretching ratios were used to obtain the total stretching ratio $\tau_{total}=V_{3}/V_{0}$. $V_{1}$ was set to induce neither stretching nor accumulation during the coagulation: $V_{1}=V_{0}$. Low, medium, and high total stretching ratios $\tau_{total}$ of 1.3, 1.5, and 1.9 were considered in the study.

*w_LMW_* (%)	Exp. Run	Coagulation		Washing		Drying		
		*V*_0_ = *V*_1_ (mm/s)	Stretch. Ratio	*V*_2_ (mm/s)	Stretch. Ratio	*V*_3_ (mm/s)	Stretch Ratio	Total Stretch. Ratio *τ_total_*
1	A	21	1	27	1.3	27	1.0	1.3
	B	21	1	27	1.3	32	1.2	1.5
	C	21	1	27	1.3	39	1.4	1.9

## Data Availability

Not applicable.
